# A Comprehensive Review of Home Sleep Monitoring Technologies: Smartphone Apps, Smartwatches, and Smart Mattresses

**DOI:** 10.3390/s25061771

**Published:** 2025-03-12

**Authors:** Bhekumuzi M. Mathunjwa, Randy Yan Jie Kor, Wanida Ngarnkuekool, Yeh-Liang Hsu

**Affiliations:** 1 Gerontechnology Research Center, Yuan Ze University, Taoyuan 320, Taiwan; 2 Mechanical Engineering Department, Yuan Ze University, Taoyuan 320, Taiwan

**Keywords:** home sleep monitoring, smartphone apps, smartwatches, smart mattresses, sleep tracking

## Abstract

The home is an ideal setting for long-term sleep monitoring. This review explores a range of home-based sleep monitoring technologies, including smartphone apps, smartwatches, and smart mattresses, to assess their accuracy, usability, limitations, and how well they integrate with existing healthcare systems. This review evaluates 21 smartphone apps, 16 smartwatches, and nine smart mattresses through systematic data collection from academic literature, manufacturer specifications, and independent studies. Devices were assessed based on sleep-tracking capabilities, physiological data collection, movement detection, environmental sensing, AI-driven analytics, and healthcare integration potential. Wearables provide the best balance of accuracy, affordability, and usability, making them the most suitable for general users and athletes. Smartphone apps are cost-effective but offer lower accuracy, making them more appropriate for casual sleep tracking rather than clinical applications. Smart mattresses, while providing passive and comfortable sleep tracking, are costlier and have limited clinical validation. This review offers essential insights for selecting the most appropriate home sleep monitoring technology. Future developments should focus on multi-sensor fusion, AI transparency, energy efficiency, and improved clinical validation to enhance reliability and healthcare applicability. As these technologies evolve, home sleep monitoring has the potential to bridge the gap between consumer-grade tracking and clinical diagnostics, making personalized sleep health insights more accessible and actionable.

## 1. Introduction

Sleep is crucial for physical, cognitive, and emotional well-being, influencing metabolic activity, immune function, cardiovascular health, and neural plasticity [1,2]. Chronic sleep deficiency has been linked to numerous health complications, including obesity, diabetes, cardiovascular disease, and mental health disorders [3]. Additionally, inadequate sleep impairs cognitive function and motor coordination, increasing the likelihood of accidents and injuries [3]. Given these risks, monitoring sleep patterns is essential for maintaining health, diagnosing sleep disorders, and improving sleep quality through behavioral or medical intervention.

Polysomnography (PSG) [4], the gold standard for diagnosing sleep disorders, records physiological parameters, such as brain activity, eye movement, muscle activity, heart rate, and breathing patterns [5]. PSG is crucial for identifying sleep-disordered breathing, excessive daytime sleepiness, and parasomnias. It is also essential for diagnosing conditions like REM sleep behavior disorder and nocturnal hypoventilation, which may indicate neurodegenerative diseases [5]. While PSG provides exceptional diagnostic detail, it is most often conducted in clinical settings due to the required specialized equipment and trained personnel [6]. As such, its use for routine or long-term sleep monitoring at home is less practical. Moreover, the in-lab setting may not always reflect a person’s typical sleep environment, potentially influencing the results. For large-scale population studies or continuous monitoring, the need for more accessible and scalable solutions has led to the development of alternative home-based sleep monitoring technologies. These alternatives aim to offer practical and cost-effective solutions without compromising the data.

This review offers a focused comparison of home-based sleep monitoring technologies, including smartphone apps, smartwatches, and smart mattresses, differentiating it from broader reviews that cover clinical and research-grade devices. While studies like [7] examine non-invasive methods, our work focuses specifically on consumer-grade solutions. We analyze 46 devices based on tracking accuracy, usability, AI-driven analytics, and healthcare integration, extending beyond systematic reviews like [8]. Our findings confirm previous concerns about accuracy, noting that consumer devices tend to overestimate total sleep time and underestimate wakefulness compared to polysomnography [9,10]. We also highlight the challenges of integrating these technologies into clinical practice, as emphasized by [11,12]. In contrast to reviews focused solely on wearables, our review includes smart mattresses and explores AI-driven multi-sensor fusion’s potential to improve reliability, echoing the perspectives of [13]. We conclude that enhanced validation methodologies and interoperability solutions are crucial for increasing the clinical applicability of consumer sleep technologies.

With the rise of remote health monitoring, understanding the reliability of consumer sleep technologies can inform both individual users and healthcare providers in managing sleep-related health conditions. Alternative home-based sleep monitoring technologies have been developed to address these limitations. These consumer-grade devices such as smartphone apps, smartwatches, and smart mattresses provide affordable, accessible, and scalable alternatives for sleep tracking [14]. Unlike PSG, these technologies allow users to monitor their sleep continuously and in real-world settings without requiring an overnight stay in a clinical lab. While they do not yet match PSG in diagnostic precision, they provide valuable insights into sleep behaviors, making them increasingly popular for general sleep health monitoring.

Recent advancements in AI-driven sleep analysis have significantly improved the accuracy of consumer sleep-tracking devices. Machine learning algorithms can analyze patterns in sleep data, distinguishing between sleep stages more effectively than traditional methods [15,16]. Additionally, AI enables real-time feedback and adaptive coaching, offering users personalized sleep improvement strategies based on long-term data trends. These innovations can bridge the gap between consumer-grade and clinical sleep monitoring by refining sleep-stage detection and enhancing the predictive capabilities of home-based sleep monitoring devices [17]. Integrating multiple sensors, such as photoplethysmography and respiratory inputs, combined with deep learning architectures, has further improved sleep-stage classification accuracy [16]. Advancements in sleep technologies include ambient room sensors, wearables, bed sensors, and mobile apps, leveraging AI for improved performance [13].

Despite these advancements, consumer sleep-tracking technologies face accuracy, validation, and standardization challenges compared to PSG. Studies have shown that these devices tend to overestimate total sleep time and underestimate wake periods [9,10,11]. Most consumer devices rely on accelerometer data, but their exact algorithms remain proprietary [11]. Consumer devices and smartphone apps demonstrated poor agreement in detecting sleep parameters, particularly in identifying wake periods compared to PSG [9]. However, some devices did better when compared to research-grade actigraphy in estimating total sleep time [10]. Researchers have proposed standardized frameworks for evaluating sleep tracker performance, including step-by-step guidelines and open-source code for data analysis to address these issues [18]. These efforts aim to enhance the reliability of consumer sleep technology and promote its informed adoption in research and clinical applications. Future research should focus on refining AI algorithms through open-source validation frameworks and incorporating multi-sensor fusion techniques to enhance reliability.

Multi-sensor fusion approaches have shown significant potential in enhancing sleep analysis and detecting anomalies in sleep patterns. By integrating data from multiple sensors, such as mmWave, UWB, piezoelectric, and 3D vision sensors, researchers have achieved higher accuracy in sleep-stage classification compared to single-sensor methods [19,20]. Additionally, incorporating physiological data from heart rate monitors, respiratory sensors, temperature sensors, and EEG-like devices enables AI algorithms to analyze and interpret complex sleep stages more effectively. A 2022 study [21] proposed a bottom-up fusion framework that integrates data-level, feature-level, and decision-level fusion to analyze sleep patterns and generate customized sleep environment recommendations. Similarly, Katz et al. [22] introduced a diffusion-based nonlinear filtering technique for multimodal data fusion, which extracts common sources of variability across sensors while filtering out sensor-specific noise. This approach has demonstrated effectiveness in sleep-stage assessment using multimodal sensor data. Overall, multi-sensor fusion techniques offer a more comprehensive perspective on sleep by capturing subtle patterns and correlations that single-sensor approaches may overlook [19,21].

Given the diversity of consumer sleep monitoring technologies, selecting the right device depends on individual needs. While some users may prioritize affordability and ease of use (e.g., smartphone apps), others may require higher accuracy and continuous physiological monitoring (e.g., smartwatches with heart rate variability (HRV) tracking). Additionally, users looking for a passive and non-intrusive option may prefer smart mattresses, which monitor sleep without requiring the user to wear a device.

### 1.1. Subsection Research Gaps and Questions

While consumer sleep technologies have gained widespread adoption, several key research gaps remain:

- Validation and Standardization: There is a lack of standardized validation methods for assessing the accuracy and reliability of consumer sleep-tracking devices compared to PSG.
- Transparency of Algorithms: The proprietary nature of many consumer-grade sleep-tracking algorithms limits their clinical validation and integration into healthcare systems.
- Integration with Clinical Settings: There is limited research on how consumer sleep technology can be effectively integrated with clinical sleep monitoring to improve diagnosis and treatment. This is due to regulatory challenges, data privacy concerns, and differences in measurement techniques.

To address these gaps, this review explores the following research questions:

1. How do consumer sleep technologies compare to PSG in terms of accuracy, reliability, and usability?
2. What are the primary limitations and challenges associated with consumer sleep-tracking devices?
3. How can AI-driven analysis and sensor technologies enhance the effectiveness of home-based sleep monitoring?

As consumer devices evolve, future research should also examine the role of hybrid models combining PSG-level accuracy with wearable convenience.

### 1.2. Research Aim

This review compares consumer sleep-tracking technologies, including smartphone apps, smartwatches, and sleep mattresses. This review seeks to provide insights into their effectiveness for personal health monitoring and applications by analyzing their features, technology, and integration with healthcare systems. The goal is to help users make informed decisions about the most suitable sleep monitoring device based on their health goals and lifestyle preferences.

The literature review for this paper was conducted using a comprehensive set of keywords, including “home sleep monitoring technologies”, “consumer sleep trackers”, “smartphone sleep apps”, “sleep monitoring smartwatches”, “smart mattress sleep tracking”, “sleep tracking devices”, “multi-sensor fusion in sleep monitoring”, “AI in sleep monitoring”, “sleep tracking accuracy”, and “consumer sleep technologies validation”. These keywords were combined with terms related to sleep disorders and clinical validation to refine the search. The research was carried out using well-established academic databases, including PubMed, IEEE Xplore, ScienceDirect, SpringerLink, and Google Scholar. These databases provided access to a wide range of studies and resources relevant to home-based sleep monitoring technologies, ensuring a thorough and up-to-date review of the field.

### 1.3. Structure of the Paper

- This review paper is structured as follows:
- Section 2: Methodology.
- Section 3: Overview of Traditional Sleep Monitoring Methods—reviews established techniques like PSG and actigraphy, providing a benchmark for evaluating consumer sleep technologies.
- Section 4: Home-Based Sleep Monitoring Technologies—examines the three major categories of consumer sleep monitoring: smartphone apps, smartwatches, and smart mattresses.
- Section 6: Comparison of Consumer Sleep Technologies—compares these devices based on data categories.
- Section 7: Discussion and future advancements in home sleep monitoring technology—identifies ongoing challenges in algorithm validation, data privacy, and interoperability while exploring potential advancements in AI and sensor technologies.
- Section 8: Conclusion—summarizes key findings and provides recommendations for future research and consumer adoption

This structured approach ensures a comprehensive analysis of home sleep monitoring technologies, guiding users in making informed choices based on their needs and preferences.

## 2. Materials and Methods

This review paper evaluates and compares home sleep monitoring technologies across three categories: smartphone apps, smartwatches, and sleep mattresses. This review focuses on sleep monitoring technologies released from 2015 onward, analyzing their usability, data categories, technology, and key features. This cutoff was chosen due to significant advancements in sensor technology, AI-driven sleep analysis, and consumer product integration, which have improved the scope and accuracy of home sleep monitoring.

### 2.1. Selection Criteria

The selection process was based on structured criteria to ensure a comprehensive and meaningful evaluation. The following factors were considered:

- Focus on Sleep Monitoring: Devices must primarily focus on sleep tracking rather than general fitness tracking.
- Availability: Devices must be commercially available on major platforms (iOS, Android, or retail channels) to ensure accessibility.
- Manufacturer Support: Excludes discontinued devices or those lacking English-language documentation.
- Smartphone App Functionality: Apps must function independently; smartwatches and smart mattresses must be integrated solutions.
- Release/Update Since 2015: Devices must be released or actively updated since 2015, reflecting technological improvements in sensor accuracy and AI analysis.
- Clinical Studies/Validation: Preference for devices supported by clinical studies or independent validation.
- AI Integration: Notes devices with AI-driven sleep analysis or integration with health ecosystems.

### 2.2. Data Collection Methods

A systematic approach combining primary and secondary sources was used to gather comprehensive data:

- Academic literature (peer-reviewed articles and clinical studies) for scientific validation and effectiveness.
- Manufacturer websites for product specifications, features, and pricing.
- App stores (Google Play, Google LLC, Mountain View, CA, USA and Apple App Store, Apple Inc., Cupertino, CA, USA) and online review platforms for real-world user feedback.
- Industry reports for insights into trends, advancements, and market dynamics.

The initial dataset included the following:

- A total of 46 smartphone applications, of which 21 met the selection criteria.
- A total of 28 smartwatches, with 16 qualifying for analysis.
- A total of 19 smart mattresses, resulting in 9 selected devices.

### 2.3. Evaluation and Analytical Approach

The devices were systematically assessed based on usability, technological components, and real-world performance. Table 1 summarizes the key evaluation metrics used:

### 2.4. Data Visualization Across Sleep Technologies

Sleep technologies use various visualization methods to enhance user engagement and for the interpretation of sleep data. The key visualization features across different device categories are summarized in Table 2.

Across all three device categories, trend analysis helps users track sleep patterns over time. Wearable devices and mobile apps provide visual breakdowns via graphs and charts, while smart mattresses also incorporate environmental data, such as temperature and humidity. Some advanced systems allow for personalized recommendations, such as AI-driven insights into optimal sleep conditions.

### 2.5. Addressing Bias and Limitations

To ensure reliability, the data were cross-referenced with independent studies and research-grade validation where available. However, the following limitations were acknowledged:

- Reliance on manufacturer claims: Some performance data were based on company-reported metrics, which may introduce bias.
- Limited access to proprietary algorithms: Many consumer-grade devices use black-box AI models, preventing independent verification of their sleep-stage classification accuracy.
- Demographic variability: Most validation studies focus on adult populations, with limited research on how these technologies perform across different age groups or individuals with sleep disorders.

## 3. Overview of Traditional Sleep Monitoring Methods

Traditional sleep assessment methods, primarily used in clinical and research settings, provide highly accurate and detailed physiological data. The two most established methods are PSG, the benchmark for evaluating newer consumer-based sleep technologies, and actigraphy. While PSG remains the gold standard, its limitations have led to the increased use of alternative methods like actigraphy, which offers a more practical approach for long-term sleep monitoring outside clinical environments.

### 3.1. Polysomnography

Polysomnography is the most comprehensive and widely accepted method for diagnosing sleep disorders. Conducted in a clinical or laboratory setting, it records multiple physiological parameters, including brain activity (EEG), eye movement (EOG), muscle tone (EMG), heart activity (ECG), breathing patterns, and oxygen saturation (SpO_2_) [5]. These measurements help determine sleep architecture and diagnose conditions, like obstructive sleep apnea (OSA), narcolepsy, REM sleep behavior disorder (RBD), and insomnia [14].

Despite its accuracy, PSG requires specialized equipment, trained personnel, and a controlled clinical setting, making it impractical for routine, long-term sleep monitoring or large-scale population studies. Furthermore, the clinical environment may sometimes alter sleep patterns, limiting its ability to reflect habitual sleep behaviors [6]. Given these factors, while PSG remains an invaluable tool for diagnosing complex sleep disorders, its use in everyday sleep monitoring at home is less feasible.

Researchers have been developing at-home PSG kits and portable EEG devices to address these challenges [23]. These innovations aim to offer clinical-grade sleep data in a more accessible and comfortable setting. While these solutions are not as widely used as traditional PSG, they represent an exciting step toward making comprehensive sleep assessments more convenient and affordable for home-based monitoring.

### 3.2. Actigraphy

Actigraphy is a non-invasive sleep monitoring technique that uses wrist-worn accelerometers to estimate sleep–wake cycles based on movement. Unlike PSG, which relies on multiple physiological signals, actigraphy primarily measures activity levels to infer sleep duration and quality. It is widely used in research and clinical practice to monitor circadian rhythm disorders, insomnia, and sleep disturbances in various populations [24].

Studies have validated actigraphy against PSG, showing moderate to high agreement for sleep duration and efficiency. However, actigraphy tends to overestimate total sleep time and underestimate wakefulness after sleep onset [25]. Its main advantage is its feasibility for long-term sleep tracking in real-world settings, making it useful for studying chronic sleep disorders and sleep–wake patterns in individuals with neurodegenerative diseases [26]. However, actigraphy cannot differentiate sleep stages, relying solely on movement detection rather than physiological signals like EEG or HRV, which limits its accuracy for diagnosing complex sleep disorders.

Recent advancements in consumer sleep technology, especially with newer smartwatches, have enhanced actigraphy by incorporating additional sensors, like HRV and oxygen saturation (SpO_2_). These additional physiological metrics help improve sleep-stage detection and overall accuracy, offering a more comprehensive approach to sleep monitoring than traditional actigraphy, which relies only on motion detection. By integrating HRV and SpO_2_, these smartwatches can better capture autonomic nervous system activity and breathing patterns during sleep, making them more capable of detecting sleep disturbances and disorders like sleep apnea [12].

### 3.3. Comparison of PSG and Actigraphy

PSG and actigraphy play critical roles in sleep assessment, but their applications differ. PSG provides detailed physiological data, making it essential for diagnosing complex sleep disorders, particularly those involving abnormal breathing patterns or neurological conditions [27]. Actigraphy, in contrast, is better suited for long-term sleep tracking, assessing behavioral sleep patterns, and evaluating the effects of lifestyle interventions [24]. While PSG offers a comprehensive analysis of sleep stages and physiological parameters, actigraphy’s strengths lie in its ability to monitor sleep over extended periods in natural settings, providing insights into sleep habits and circadian rhythms.

To provide a clear comparative overview, a table summarizing the differences between PSG and actigraphy in terms of various factors such as accuracy, data collected, sleep-phase detection, application, and limitations is included, as illustrated in Table 3.

Advancements in wearable technology and artificial intelligence (AI) are helping bridge the gap between PSG and actigraphy by improving non-invasive sleep-tracking methods. Many modern consumer sleep devices incorporate heart rate sensors, motion analysis, and AI-driven sleep staging algorithms to enhance accuracy [12]. While they do not yet match PSG in diagnostic precision, these innovations offer promising alternatives for continuous, real-world sleep monitoring. As research progresses, the integration of multi-sensor tracking, AI-driven analysis, and clinical validation may lead to more reliable, accessible, and cost-effective solutions for improving sleep health.

## 4. Home-Based Sleep Monitoring Technologies

Home-based sleep monitoring technologies have emerged as viable alternatives to traditional clinical assessments, offering individuals convenient and continuous sleep-tracking solutions. Unlike PSG, which requires overnight stays in specialized sleep laboratories, home-based technologies enable users to monitor their sleep in familiar environments, improving adherence and real-world applicability [14]. These consumer-grade devices leverage sensor technologies and data analysis techniques to estimate sleep parameters, making them increasingly popular among the general population and healthcare professionals seeking scalable sleep assessment tools [28].

Home-based sleep monitoring technologies can be classified into three major categories: smartphone applications, wearable devices (smartwatches), and smart mattresses. Each technology employs distinct methods for tracking sleep patterns, ranging from motion sensors and heart rate monitoring to advanced artificial intelligence (AI) algorithms for sleep-stage classification [13]. Table 4 summarizes the main categories of home-based sleep monitoring technologies, highlighting the data they collect, their primary applications, and their limitations. Each category offers unique benefits for different sleep monitoring needs, from general sleep tracking to in-depth health analysis.

### 4.1. Sleep Monitoring Using Smartphone Applications

Smartphone-based sleep monitoring apps utilize built-in sensors, such as accelerometers and microphones, to track sleep duration, disturbances, and snoring patterns [29,30]. These applications analyze motion and sound data to estimate sleep onset, wakefulness, and efficiency. Advanced applications integrate AI-driven sleep coaching, providing users with personalized feedback based on long-term sleep patterns [31].

One of the major strengths of smartphone-based sleep monitoring is its high accessibility. These apps are available on iOS and Android, requiring no additional hardware, making sleep tracking widely accessible. Additionally, they are cost-effective, with many apps being free or low-cost compared to other sleep monitoring devices. Their ease of use further enhances their appeal, as they require minimal setup compared to wearables or PSG. Some apps also provide snore and noise detection, leveraging microphone-based tracking to help identify potential sleep apnea symptoms [32].

**Figure 1:**
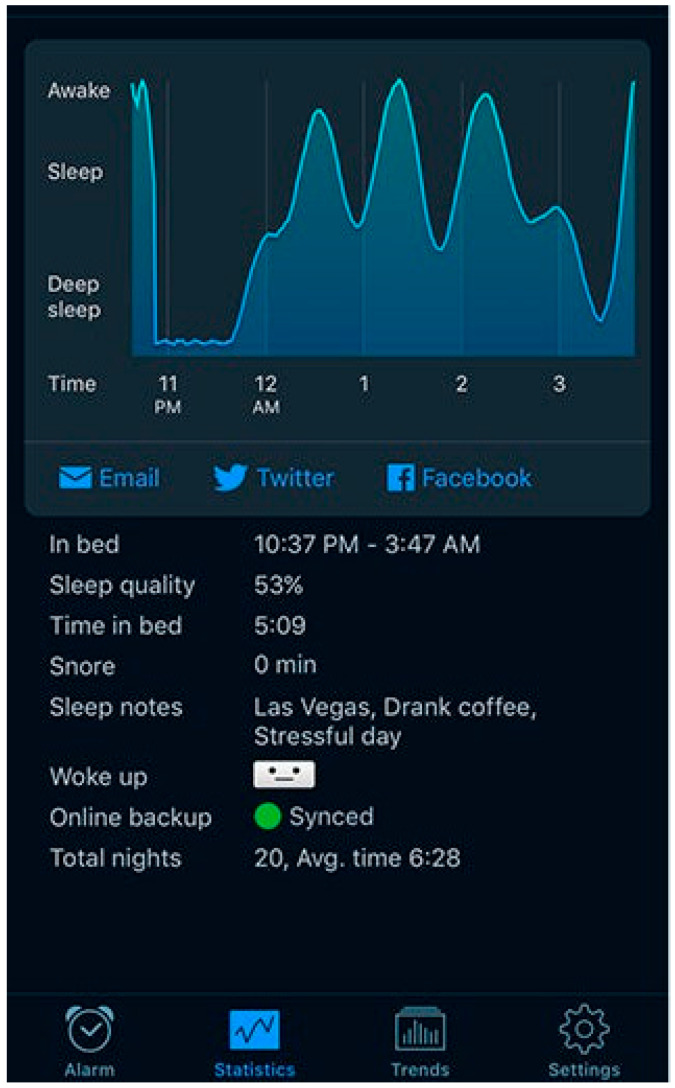
Example mobile app [33].

Different smartphone apps offer varying levels of sleep-tracking functionality. While some focus primarily on snoring detection and smart alarms, others provide AI-driven sleep insights and personalized recommendations. Figure 1 illustrates the interface of a smartphone sleep monitoring app, showcasing sleep tracking results.

To ensure a comprehensive review of smartphone-based sleep monitoring applications, the following selection criteria were applied:

1. The app must function independently without requiring external sensors or hardware.
2. The app must track core sleep-tracking features including sleep duration, quality, and disturbances, rather than focusing solely on relaxation or meditation.
3. The app should be available on either iOS, Android, or both.
4. Apps with high user engagement, positive reviews, and substantial download counts.
5. Apps with research-backed algorithms or clinical studies were favored whenever possible.
6. Only actively maintained and updated apps were included to ensure relevance.

Based on these criteria, an initial review of 46 smartphone applications was conducted. Of these, 21 apps met the selection requirements and were included in the final analysis. Table 5 summarizes the key features of the selected smartphone sleep-tracking applications.

#### 4.1.1. Limitations of Smartphone-Based Sleep Monitoring

Despite their accessibility and cost-effectiveness, smartphone sleep-tracking apps have notable limitations. These apps rely on indirect measurements, which can be affected by external noise, improper device placement, and environmental factors, leading to reduced accuracy in sleep staging compared to clinical methods [55]. Additionally, consumer sleep-tracking applications vary widely in effectiveness, usability, and integration with healthcare platforms. Some apps, such as Sleep Cycle and SnoreLab, provide detailed sleep reports and smart alarms, while others, like AutoSleep and Pillow, integrate smartwatch data for enhanced accuracy [56].

Given these limitations, ongoing research seeks to enhance smartphone-based sleep monitoring through improved algorithms and multi-sensor integration.

#### 4.1.2. Research on Smartphone-Based Sleep Monitoring

Research on smartphone-based sleep monitoring has primarily focused on evaluating accuracy, identifying limitations, and enhancing detection methods. While these apps provide a convenient way to track sleep patterns, their reliability compared to clinical gold standards like PSG varies significantly. Comparative studies indicate that smartphone apps estimate sleep duration reasonably well but tend to overestimate sleep efficiency and underestimate wakefulness [57]. As a result, while useful for general sleep habit tracking, they cannot be used for clinical sleep disorder diagnoses. Specific app evaluations demonstrate these variations in accuracy. For example, Snorefox M showed high sensitivity (0.91) and specificity (0.83) for detecting moderate to severe obstructive sleep apnea in a non-symptomatic population [58]. Sleep Time, on the other hand, exhibited a poor correlation with PSG in measuring sleep efficiency, light sleep percentage, and deep sleep percentage despite achieving high sensitivity (89.9%) in detecting sleep [59]. These findings highlight the inconsistent reliability of smartphone-based sleep monitoring across different applications.

To improve the accuracy and functionality of smartphone sleep monitoring, current research explores several key advancements: leveraging machine learning to enhance sleep-stage classification; combining smartphone audio with smartwatch HRV data for more precise sleep analysis; developing improved algorithms to better differentiate sleep phases; and implementing adaptive soundscapes, light adjustments, and biofeedback techniques to promote sleep quality [56]. By incorporating these innovations, smartphone-based sleep monitoring is evolving into a more reliable tool for long-term sleep health tracking, with the potential for integration into digital healthcare ecosystems.

### 4.2. Sleep Monitoring Using Smartwatches

Wearable sleep-tracking devices serve as an intermediary between smartphone-based applications and clinical PSG, offering a balance of accuracy, accessibility, and convenience [60]. Unlike smartphone applications that primarily rely on motion and audio-based estimations, wearable devices continuously capture biometric parameters, yielding more detailed insights into sleep architecture.

These devices, which include smartwatches, fitness bands, and smart rings, have gained widespread adoption for their ability to monitor physiological signals during sleep [61]. By integrating photoplethysmography (PPG) sensors, accelerometers, and gyroscopes, wearables can measure HRV, body movement, respiratory patterns, and blood oxygen saturation (SpO_2_). More advanced models incorporate AI-powered sleep analysis, refining sleep-stage classification and enhancing the differentiation between light sleep, deep sleep, and rapid eye movement (REM) sleep [62].

Compared to smartphone-based sleep monitoring, wearable devices provide continuous biometric tracking, allowing for a more comprehensive assessment of sleep architecture [60]. While accelerometer-based wearables effectively distinguish between sleep and wake states, the inclusion of PPG-derived data significantly improves sleep-stage classification accuracy [63]. However, consumer-grade wearables remain susceptible to limitations, including a tendency to overestimate total sleep time and efficiency when compared to gold-standard PSG assessments [11].

**Figure 2:**
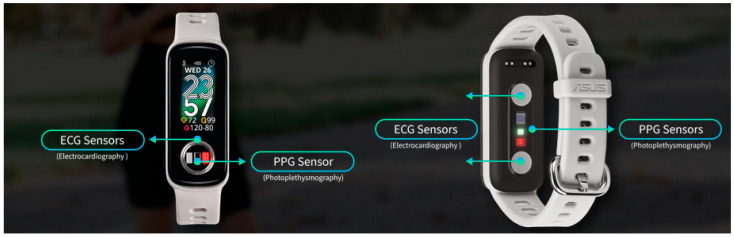
Example of sleep monitoring wearable device [64].

To enhance the reliability of wearable sleep-tracking devices, researchers recommend validating algorithms across diverse populations, standardizing performance metrics, and promoting open-source classifiers and datasets [63]. Despite their limitations, these devices offer a practical compromise between accessibility and accuracy, making them valuable tools for long-term sleep monitoring outside clinical settings [60] (see Figure 2).

#### 4.2.1. Selection Criteria for Wearable Devices

The selection of wearable sleep-tracking devices for this review was based on a structured set of criteria designed to ensure a comprehensive and meaningful evaluation. Initially, 30 smartwatches were reviewed, of which 16 met the selection criteria. Market availability and user adoption were primary considerations, with preference given to devices that have garnered widespread consumer recognition and consistently positive reviews. Only wearables that are currently available on the market were included to maintain relevance.

Feature comprehensiveness was another key criterion; selected devices had to incorporate essential sleep-tracking functionalities, including HRV monitoring, SpO_2_ measurement, sleep-stage tracking, and motion detection. Preference was given to wearables integrating AI-driven sleep analysis, given their potential for enhanced accuracy and advanced sleep architecture modeling.

Furthermore, integration with broader health ecosystems was prioritized. Devices compatible with Apple Health, Google Fit, and electronic health record (EHR) systems were favored, as they facilitate seamless data exchange and enable remote health monitoring. Lastly, scientific validation and clinical relevance were integral to the selection process. Devices with peer-reviewed studies evaluating their accuracy were included, while those lacking publicly available validation data were excluded.

#### 4.2.2. Key Features of Wearable Sleep Trackers

Wearable sleep monitors offer a diverse range of functionalities, spanning from basic sleep tracking to advanced physiological monitoring. Table 6 presents a comparative analysis of the key features of the selected wearable sleep-tracking devices.

#### 4.2.3. Strengths and Limitations of Wearables

Wearable sleep trackers offer several advantages over smartphone-based monitoring. A key benefit is their higher accuracy, as they continuously track HRV, SpO_2_, and movement, leading to more precise sleep staging [81]. They also enable long-term sleep trend analysis, which is valuable for tracking chronic sleep patterns and detecting potential disturbances over time. Many wearables integrate with health ecosystems, including Apple Health, Google Fit, and telemedicine platforms, facilitating remote monitoring by healthcare professionals. Additionally, wearables are portable and non-invasive, allowing users to conduct sleep monitoring at home rather than in a clinical setting.

Despite these advantages, wearable sleep-tracking devices face notable limitations. REM sleep detection remains a challenge, as movement-based algorithms often misclassify REM as light sleep, leading to inaccuracies in sleep architecture analysis [56]. Sensor accuracy is another issue, as wrist-worn devices can misinterpret hand movements as wakefulness, reducing the precision of sleep-stage classification [82]. Additionally, some users experience discomfort wearing devices overnight, which can reduce adherence to long-term monitoring. Another limitation is that many wearables rely on proprietary algorithms, making independent validation difficult and raising concerns about data transparency and accuracy [13]. Device cost can be influenced by other functions that are not related to sleep monitoring.

#### 4.2.4. Clinical Validation and Research Insights for Wearable Devices

Research studies comparing wearable devices to gold-standard PSG testing have reported mixed accuracy results. For instance, a study on the Oura Ring found an 80% agreement with PSG for total sleep time but significant misclassification of deep and REM sleep [81]. Similarly, comparisons of Fitbit and Apple Watch models indicate 70–85% agreement in measuring total sleep duration, yet their performance in accurately distinguishing sleep stages varies [83].

These findings highlight the need for further advancements in sensor accuracy and machine learning models to enhance sleep staging. Future research aims to improve REM sleep detection through multi-sensor fusion approaches, combining HRV, respiratory rate, and motion data for more reliable classification. Another emerging area is AI-driven sleep analysis, where machine learning algorithms refine sleep tracking by learning from large-scale sleep datasets. Additionally, the development of more comfortable and lightweight wearables could improve long-term user compliance, making these devices more viable for continuous sleep health monitoring.

### 4.3. Sleep Monitoring Using Smart Mattresses

Smart mattresses and under-mattress sleep-tracking systems provide a passive, non-intrusive alternative to wearable sleep monitors. Unlike wrist-worn or ring-based trackers, these systems do not require direct skin contact, making them particularly beneficial for individuals who experience discomfort with traditional wearables.

**Figure 3:**
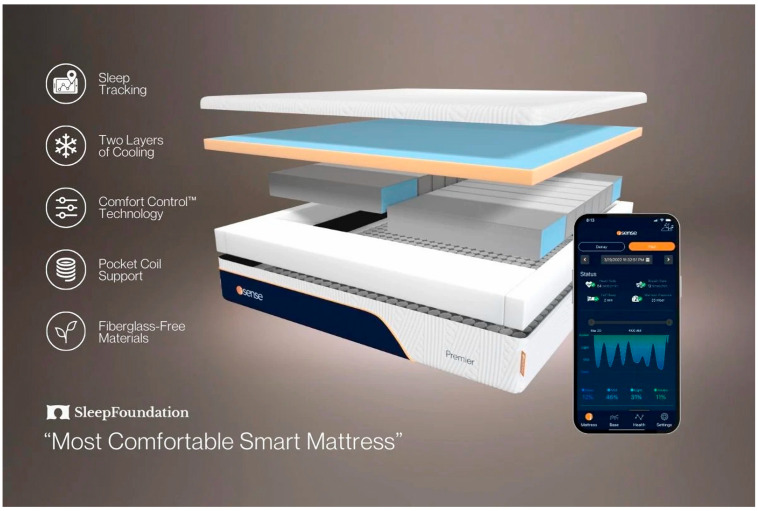
Example of sleep monitoring smart mattress [85].

These systems leverage advanced sensing technologies such as ballistocardiography (BCG), fiber-optic sensing, and pressure mapping to monitor key sleep metrics, including heart rate (HR), respiratory rate (RR), body movement, and sleep duration [84]. Some smart mattresses also integrate AI-driven sleep analysis, offering detailed insights into sleep efficiency and disturbances. Their ability to provide continuous, contactless sleep tracking makes them an attractive option for long-term sleep monitoring at home (see Figure 3).

#### 4.3.1. Selection Criteria for Smart Mattresses

To ensure a thorough and meaningful comparison, this review initially examined 19 smart mattresses. After applying the selection criteria, nine devices were included in the final analysis. The selection process was guided by the following factors:

- Technological Sophistication—Devices needed to incorporate multi-sensor capabilities, including HR and RR monitoring, sleep-stage detection, and body movement analysis. Preference was given to mattresses featuring AI-driven insights and automatic sleep optimization adjustments.
- Market Availability and User Adoption—Only commercially available smart mattresses with established consumer use were included to ensure practical relevance.
- Integration with Health and Smart Home Ecosystems—Priority was given to devices that sync with mobile apps, wearable devices, or broader health-tracking platforms, enabling seamless data sharing and enhanced sleep management.
- Clinical Validation and Accuracy—Smart mattresses supported by peer-reviewed studies or manufacturer-provided validation reports were favored, while those lacking publicly available validation data were excluded.

#### 4.3.2. Key Features of Smart Mattresses and Under-Mattress Sensors

Smart mattresses and non-wearable sleep trackers offer various features to enhance sleep monitoring and improve sleep quality. Table 7 presents a summary of selected devices and their primary functionalities.

#### 4.3.3. Strengths and Limitations of Smart Mattresses

Smart mattresses offer several advantages over wearable and smartphone-based sleep tracking. One of their primary strengths is non-intrusive and passive monitoring, making them particularly beneficial for individuals who find wearable devices uncomfortable. Since these systems track physiological signals without requiring direct body contact, they provide a seamless, long-term solution for sleep tracking without affecting natural sleep behavior. Additionally, smart mattresses tend to be more reliable than wearables for detecting sleep onset and wake after sleep onset (WASO), improving accuracy in sleep duration estimation [94].

However, sensor-integrated mattresses also present notable limitations. One key challenge is interference from co-sleepers, as shared beds introduce measurement errors, affecting data precision [95]. Unlike personal wearables that track individual users, smart mattresses may struggle to differentiate between two occupants, reducing their effectiveness for multi-user environments. Additionally, these systems are often more expensive than wearables and smartphone-based trackers, limiting their accessibility for many consumers. While they provide high-quality, continuous sleep tracking, their cost and potential accuracy issues with co-sleepers remain barriers to wider adoption.

#### 4.3.4. Clinical Validation and Research Insights for Smart Mattresses

Studies evaluating sensor-integrated mattresses have demonstrated promising accuracy when compared to gold-standard PSG. Research by Walsh and McLoone (2014) [96] found that smart mattresses achieve an 85–90% agreement with PSG for total sleep time estimation, validating their effectiveness for long-term home-based sleep tracking.

Despite this high level of agreement, smart mattresses are not suitable for diagnosing complex sleep disorders, such as sleep apnea or REM behavior disorder, which require detailed neurophysiological data [83]. Instead, these devices serve as useful tools for monitoring long-term sleep trends, identifying irregular sleep patterns, and optimizing sleep environments.

Recent advancements in AI-powered sleep analysis and non-contact biosensors may further improve data accuracy and usability. Future innovations are expected to enhance co-sleeper differentiation, affordability, and integration with digital health ecosystems, making smart mattresses a more viable option for broad consumer adoption.

## 5. Technology for Different Scenarios

Sleep monitoring technology varies significantly in usability, accuracy, and suitability depending on user needs. This section explores different sleep-tracking solutions tailored for general users, individuals with sleep disorders, athletes, elderly populations, mental health support, and children’s sleep monitoring, assessing their effectiveness and challenges.

### 5.1. General Usage

For individuals seeking to improve sleep quality and ensure restorative rest, sleep tracking provides essential insights. The National Sleep Foundation (NSF) developed age-based sleep duration guidelines, emphasizing the importance of adequate deep and REM sleep for physical and cognitive restoration [97]. Sleep efficiency, the percentage of time spent asleep relative to time in bed, is a key health measure, with low sleep efficiency (<80%) linked to increased mortality risk in older adults [98]. Modern sleep-tracking solutions, such as wearable devices (e.g., Fitbit and Apple Watch) and non-wearable sensors (e.g., under-mattress sensors and radar-based bedside monitors), provide real-time data on sleep patterns, WASO (wake after sleep onset), and sleep consistency. Variability in sleep patterns has been associated with chronic low-grade inflammation, as indicated by increased white blood cell counts [99]. By leveraging these technologies, individuals can optimize their sleep for better overall health and well-being.

### 5.2. Individuals with Sleep Disorders

Sleep disorders affect 50–70 million Americans, including conditions such as insomnia, sleep apnea, circadian rhythm disorders, and parasomnias [100]. Insomnia and obstructive sleep apnea (OSA) are among the most prevalent and often co-occur as Comorbid Insomnia and Sleep Apnea (COMISA) [101]. Tracking sleep data is essential for diagnosis and management, as insomnia is associated with altered glucose and amino acid metabolism, while OSA affects lipid metabolism [102]. Medical-grade sleep monitoring solutions, such as at-home PSG (e.g., WatchPAT and ResMed ApneaLink), provide clinical-grade data by monitoring oxygen saturation, breathing irregularities, heart rate, and WASO. A study of U.S. veterans found a six-fold increase in sleep disorder diagnoses from 2000 to 2010, with sleep apnea (47%) and insomnia (26%) being the most common [103]. Such technologies help detect early symptoms and guide treatment strategies.

### 5.3. Athletes and High-Performance Users

Athletes and fitness enthusiasts utilize sleep tracking to optimize recovery and enhance performance. Sleep supports physical development, emotional regulation, and cognitive functioning, all of which are critical for athletic success. Research indicates that elite athletes typically achieve 7–8 h of sleep but may experience increased WASO due to training schedules or stress [104,105].

Wearable sleep trackers (e.g., WHOOP and Oura Ring) provide in-depth analytics on sleep stages, HRV, and recovery scores. Smart mattresses (e.g., Eight Sleep Pod and Sleep Number 360) adjust temperature and firmness to improve sleep quality. Studies show that monitoring sleep patterns can reduce injury risk, improve recovery, and enhance training consistency [106]. These technologies allow athletes to tailor routines for peak performance [107].

### 5.4. Monitoring Sleep for Mental Health

Sleep tracking plays a crucial role in mental health, especially for individuals dealing with anxiety, depression, and stress-related disorders. REM sleep is essential for emotional regulation and the formation of adaptive stress responses [108]. Disruptions in sleep, such as prolonged sleep latency and irregular patterns, are linked to increased anxiety, depression, and other psychological challenges [109,110].

AI-powered sleep monitoring (e.g., SleepScore Max and bedside radar monitors) and wearables track HRV, a key marker of mental well-being. Higher HRV is correlated with better sleep quality and fewer nighttime awakenings, making it a valuable metric for mental health assessment [111,112]. Personalized insights from these technologies can aid in stress reduction and behavioral interventions.

### 5.5. Parents Monitoring Children’s Sleep

Parents monitor their children’s sleep to ensure healthy development and address issues such as nightmares or night terrors. Sleep duration recommendations vary by age, with infants requiring 12–16 h and teenagers needing 8–10 h per day [113]. REM sleep contributes to emotional regulation and memory integration, while NREM sleep aids memory consolidation [114].

AI-based baby monitors (e.g., Nanit and Owlet) track breathing, movement, and environmental factors, such as room temperature, noise, and light. Frequent awakenings may indicate discomfort or sleep disorders [115]. By analyzing sleep patterns, these technologies help parents create an optimal sleep environment for improved sleep quality and overall well-being [116].

### 5.6. Elderly and Accessibility Considerations

Sleep disturbances in older adults are increasingly recognized as important indicators of cognitive decline and cardiovascular health risks. Studies have shown that irregular sleep patterns are associated with increased risks of cardiovascular disease, obesity, hypertension, and diabetes [117]. Sleep irregularity has also been linked to higher all-cause mortality rates [118]. Non-wearable, contactless solutions (e.g., under-mattress sensors and radar-based monitors) are ideal for elderly care, offering passive and continuous monitoring without requiring active user engagement. Technologies like the Withings Sleep Analyzer and Google Nest Hub use sound and motion tracking to assess sleep efficiency, breathing patterns, and nighttime disturbances. Such solutions help caregivers and medical professionals detect early warning signs and adjust care plans accordingly.

By tailoring sleep-tracking technologies to specific needs and scenarios, individuals can optimize sleep quality, address potential issues, and enhance overall health and performance. Advances in AI, wearable sensors, and contactless monitoring are transforming how sleep is monitored, making personalized interventions more accessible and effective.

### 5.7. Comparison of Sleep Monitoring Technologies by Scenario

Different user groups require sleep monitoring technologies tailored to their specific needs. General users benefit from smartphone apps and wearables that provide basic sleep insights at an affordable cost, while individuals with sleep disorders may require FDA-approved devices for more accurate tracking. Athletes prioritize HRV analysis and deep sleep monitoring to optimize recovery, whereas elderly individuals benefit from passive, non-contact sensors that provide caregiver alerts. Mental health applications use HRV-based stress analysis and AI-driven sleep coaching, and children’s sleep monitoring focuses on safety alerts and sleep duration tracking. Table 8 provides a comparative summary of sleep monitoring technologies across different user scenarios.

## 6. Comparison Across Technology Categories

The field of sleep-tracking technologies offers diverse devices and solutions, each tailored to meet varying user needs. These technologies differ significantly in their tracking methods, data precision, integration with other devices, user interface and experience, and additional features. This section delves into the trade-offs between cost, accuracy, and user experience, along with a detailed comparison of different devices categorized as sleep apps, smartwatches, and smart mattresses.

### 6.1. Cost–Benefit Analysis of Home Sleep Monitoring Devices

The cost–benefit analysis of home sleep monitoring devices highlights key differences in affordability, accuracy, and health benefits among smartphone apps, wearables, and smart mattresses. Smartphone apps are the most cost-effective option (free to ~USD50); however, their algorithms lack scientific validation, limiting their clinical utility [30,55]. Wearables, such as smartwatches and smart rings, offer improved accuracy by integrating physiological monitoring (e.g., HRV and SpO_2_) and AI-driven coaching, making them ideal for athletes and sleep-conscious individuals. Despite their advantages, wearables face challenges, including accuracy, comfort, data privacy, and regulatory hurdles [119,120]. Smart mattresses, the most expensive option (USD500–USD3000), provide passive, non-intrusive sleep tracking using BCG and temperature sensors, making them beneficial for long-term users. While most systems focus on individual monitoring, some innovations now allow for the simultaneous tracking of two individuals on a single mattress [121]. Overall, wearables offer the best balance of cost, accuracy, and health benefits, while smartphone apps cater to budget-conscious users, and smart mattresses are best suited for those seeking long-term, hands-free monitoring (see Table 9).

### 6.2. Trade-Offs Between Cost, Accuracy, and User Experience

When evaluating sleep-tracking devices, users often face trade-offs between cost, accuracy, and user experience. High-end devices like the Oura Ring or Whoop Strap provide highly accurate data on sleep stages, heart rate, and body temperature but are costly. More affordable solutions, such as SleepBot or Sleep++, offer basic insights but may lack precision. While expensive devices often provide seamless integration, detailed reports, and advanced features like real-time coaching, budget-friendly, non-wearable options like StaySleep prioritize accessibility and ease of use without requiring additional hardware.

Wearable devices typically deliver data that are more accurate but can be uncomfortable, which may deter consistent usage. In contrast, non-wearable options such as Pillow and Sleep Monitor emphasize comfort and simplicity, though their accuracy may be limited compared to high-end wearables. Users must weigh these trade-offs to choose a device that aligns with their preferences and priorities.

### 6.3. Comparing Collected Data Types Across Device Categories

Sleep-tracking technologies vary in their ability to capture data across four key categories: sleep metrics, physiological data, movement data, and environmental data. Table 10 below summarizes the capabilities of sleep apps, smartwatches, and smart mattresses in these areas:

This analysis highlights the diverse capabilities of devices. For instance, sleep apps like BetterSleep provide personalized insights for improving sleep quality, while devices like Fitbit Charge 6 and Sleep Number 360 monitor sleep disorders, such as apnea. Smartwatches, including the Apple Watch Series 9 and Garmin Venu 3S, integrate sleep tracking with fitness and health data, offering a holistic wellness approach.

### 6.4. Applications and Use Cases

Sleep-tracking technologies support a wide range of applications, from personalized sleep management to lifestyle integration:

- Personalized Sleep Management: Apps like BetterSleep and devices such as Oura Ring provide tailored recommendations to help users improve sleep quality.
- Sleep Disorder Monitoring: Devices like the Sleep Number 360 and Fitbit Charge 6 are valuable for tracking conditions, such as sleep apnea or restless sleep patterns.
- Lifestyle Integration: Smartwatches like the Apple Watch Series 9 and Garmin Venu 3S combine sleep tracking with fitness and overall health monitoring, promoting a balanced lifestyle.

The variety of features and price points ensures users can select a device that meets their specific needs, whether focused on affordability, accuracy, or user experience. Understanding these factors is crucial for making informed decisions. For a more detailed breakdown, refer to the appendix for an in-depth comparison of individual devices.

### 6.5. AI-Based Sleep-Stage Classification

Many modern sleep monitoring devices use AI-driven sleep-tracking algorithms to improve accuracy. However, AI performance differs by category:

- Smartphone apps primarily use sound and motion data, making their AI models more prone to false detections from external noises and movement.
- Wearables integrate HRV, motion, and respiration signals, allowing AI models to refine sleep-stage detection.
- Smart mattresses use ballistocardiography (BCG), which tracks subtle body movements, but AI models for these systems require calibration to avoid interference from bed sharing and external vibrations.

### 6.6. Choosing the Right Technology for Different Scenarios

Selecting the most suitable sleep monitoring technology depends on the user’s needs, lifestyle, and health considerations:

- General Users: Smartphone apps provide a low-cost and accessible solution for those who want basic sleep duration tracking but lack advanced physiological data collection.
- Athletes and Individuals with Sleep Disorders: Wearables (e.g., smartwatches and fitness trackers) provide HRV, respiratory rate, and motion-based sleep staging with higher accuracy than smartphone apps.
- Elderly Individuals or Passive Monitoring Users: Smart mattresses offer an unobtrusive way to track sleep duration and physiological parameters like heart rate and breathing patterns without requiring a wearable device.
- Mental Health Applications: Wearables with HRV-based stress analysis (e.g., Oura Ring, Oura Health Oy, Oulu Finland and WHOOP, Boston, MA, USA) and AI-driven sleep coaching apps (e.g., Sleepio, Big Health, London, UK and Calm, Calm.com, San Francisco, CA, USA) provide valuable insights into the relationship between sleep quality and emotional well-being.
- Parents Monitoring Children’s Sleep: Smart baby monitors (e.g., Nanit, New York, USA and Owlet, Lehi, UT, USA) provide real-time safety alerts and oxygen-level tracking, offering peace of mind.

By referencing Table 10, users can identify the best technology category suited for their specific sleep-tracking goals and comfort preferences.

## 7. Discussion and Future Advancements in Home Sleep Technology

Home sleep monitoring technology has significantly advanced in recent years, offering greater accessibility and convenience for users. However, accuracy, clinical validation, AI integration, and data privacy remain critical areas for improvement. This section discusses the current state of sleep-tracking technologies and the future advancements that can enhance their reliability and effectiveness.

### 7.1. Current Strengths and Limitations

Home sleep monitoring technologies provide accessible and cost-effective solutions for tracking sleep patterns outside clinical settings. Smartphone applications are the most affordable option, leveraging built-in sensors like accelerometers and microphones to estimate sleep duration and detect disturbances. Wearable devices, including smartwatches and fitness bands, enhance sleep tracking by continuously monitoring physiological signals such as HRV and blood oxygen saturation (SpO_2_), offering a more detailed assessment of sleep architecture [61]. Smart mattresses provide passive, non-intrusive monitoring through ballistocardiography (BCG) and pressure sensors, making them suitable for long-term sleep tracking without requiring direct user interaction [62].

These technologies empower users by providing personalized insights into their sleep habits, promoting lifestyle adjustments, and integrating with health platforms like Apple Health and Google Fit. Such integration facilitates a more holistic approach to sleep health and may contribute to early detection of sleep disorders [56].

The long-term use of home-based sleep technologies can have both positive and negative impacts on sleep health and overall well-being. Consumer sleep technologies (CSTs), including mobile apps, wearables, and embedded devices, offer valuable insights into sleep patterns and behaviors, potentially improving sleep hygiene and facilitating behavioral interventions [122]. These technologies can support personalized sleep education, enhance self-awareness, and aid in establishing consistent sleep routines, particularly for families and children [123]. For older adults, continuous sleep monitoring through wearables and non-wearable devices can help identify sleep disturbances and their associations with health conditions, enabling proactive management of sleep-related disorders [124]. Key parameters such as total sleep time, wake after sleep onset, and sleep efficiency provide objective data that can contribute to long-term sleep health improvements [124]. However, the widespread adoption of CSTs also raises concerns regarding data accuracy, over-reliance on technology, and potential sleep anxiety caused by excessive self-monitoring. Additionally, the integration of CSTs into clinical sleep medicine remains an ongoing challenge, requiring validation and standardization to ensure their reliability and clinical applicability [122]. Despite these challenges, the advancements in CSTs present significant opportunities for large-scale sleep research and real-world sleep health assessment, contributing to broader public health initiatives [12].

Despite these advantages, limitations persist. Smartphone-based applications rely on indirect estimation methods, which can be affected by environmental noise and improper device placement. Wearable devices offer better precision but may struggle with REM sleep classification and can be uncomfortable for overnight use [11]. Smart mattresses, while convenient, face challenges in distinguishing between co-sleepers and maintaining measurement accuracy in different sleeping positions [63]. Furthermore, proprietary sleep-tracking algorithms limit transparency, making it difficult to compare devices or integrate them into clinical workflows. Addressing these issues through improved sensor technologies, standardized validation protocols, and AI-driven sleep assessment models is crucial for enhancing the reliability of home-based sleep monitoring.

### 7.2. Accuracy and Validation Challenges

The accuracy of consumer sleep monitoring technologies varies significantly compared to the gold-standard PSG. Smartphone-based applications primarily use accelerometers and microphones, but studies show they tend to overestimate total sleep time and underestimate wakefulness [57]. Device placement significantly influences accuracy, as placing a smartphone on a nightstand yields more precise data than placing it under a pillow, where sensor sensitivity may be reduced [125,126]. Wearable devices improve accuracy by incorporating HRV and SpO_2_ sensors, yet they still struggle with precise sleep-stage classification, particularly in distinguishing between light, deep, and REM sleep [60]. Smart mattresses provide passive monitoring through BCG and pressure sensors, demonstrating better accuracy in detecting sleep onset and duration. However, they face limitations in multi-user environments, as co-sleepers can introduce signal interference [62].

One key challenge is the lack of transparency in proprietary sleep-tracking algorithms, which prevents independent verification of their accuracy [58]. Unlike PSG, which follows standardized clinical guidelines, many commercial devices rely on black-box machine learning models trained on undisclosed datasets, complicating comparisons across devices. Efforts to address this include the following: (1) developing open-source benchmarking tools to assess sleep-tracking accuracy, (2) conducting large-scale comparative studies against PSG, and (3) exploring multi-sensor fusion, combining actigraphy, HRV, and respiratory data to enhance classification accuracy. Future research should refine validation methodologies, ensuring consumer sleep technologies align more closely with clinical standards while maintaining user accessibility.

Well-known consumer sleep trackers, such as the Fitbit Charge 2, Fitbit Alta HR, Garmin Vivosmart 4, Oura Ring Gen 2, and Withings Sleep Tracking Mat, use proprietary algorithms to monitor sleep patterns. These algorithms are crucial for device functionality, analyzing data collected from sensors to estimate sleep stages and quality. However, the proprietary nature of these algorithms poses significant challenges for independent validation. Researchers and clinicians cannot access the specific computational methods and raw data used, which limits the ability to independently verify the accuracy and reliability of the sleep data provided by these devices. For instance, studies have shown that devices like the Fitbit and Garmin Vivosmart 4 may overestimate sleep duration and underestimate wake periods, particularly in clinical populations, such as individuals with obstructive sleep apnea [127]. This limitation in transparency affects not only clinical reliance but also cross-comparison among devices, as each uses different underlying methodologies to interpret similar physiological signals [10,127,128,129,130,131].

### 7.3. Integration with Healthcare Systems

Integrating home sleep monitoring technologies with healthcare systems presents both opportunities and challenges. Consumer sleep-tracking devices provide valuable insights, but their clinical adoption is hindered by the following:

- Data Accuracy and Standardization: Many consumer devices do not meet the rigorous formatting standards required for clinical decision-making [56].
- Proprietary Algorithms: A lack of transparency prevents healthcare providers from verifying the reliability of these devices [59].
- Regulatory Compliance: Ensuring compliance with HIPAA (U.S.) and GDPR (Europe) remains a significant challenge.

Standardized frameworks, such as HL7 FHIR (Fast Healthcare Interoperability Resources), offer potential pathways for integrating consumer sleep data with electronic health records (EHRs). Additionally, advancements in AI-driven analysis and multi-sensor fusion could enhance the reliability of consumer devices, making them more suitable for clinical applications. Collaboration between technology developers, medical institutions, and regulatory bodies will be essential to validate these devices through large-scale clinical trials. If successfully integrated, home-based sleep monitoring could enable the early detection of sleep disorders, personalized treatment plans, and continuous remote monitoring.

### 7.4. The Role of AI and Machine Learning

Artificial intelligence (AI) and machine learning have significantly improved home sleep monitoring technologies by enhancing sleep-stage classification and enabling real-time sleep tracking. Traditional consumer sleep trackers primarily relied on accelerometers, which often misclassified wake periods and sleep stages. In contrast, AI-driven models leverage multi-sensor fusion, incorporating HRV, respiratory patterns, and SpO_2_ data for improved accuracy [56].

Multi-sensor fusion in home sleep monitoring enhances accuracy by integrating data from multiple sensors, allowing AI models to provide more precise sleep-stage classification and detect sleep disturbances more effectively. Commonly used sensors include photoplethysmography (PPG) for heart rate variability (HRV) and oxygen saturation [16], accelerometers and gyroscopes for motion tracking [12], and ballistocardiography (BCG) for non-intrusive heart rate and respiratory monitoring in smart mattresses [18]. Additional sensors such as respiratory monitors, electrodermal activity (EDA), microphones, and temperature sensors contribute to detecting breathing irregularities, stress levels, environmental disturbances, and sleep apnea [17,132,133]. AI-driven deep learning models, particularly convolutional and recurrent neural networks (CNNs and RNNs), process these multimodal inputs to differentiate sleep stages more accurately and minimize false detections. Studies show that integrating multiple sensor streams improves REM sleep detection accuracy by up to 30% compared to single-sensor approaches [132]. Future research should focus on refining multi-sensor fusion frameworks and optimizing AI algorithms for greater reliability in home-based sleep monitoring [18].

Recent advancements in deep learning, particularly convolutional neural networks (CNNs) and recurrent neural networks (RNNs), have significantly improved sleep-stage classification in consumer sleep monitoring devices [58]. These models analyze multi-sensor data, including PPG, respiratory signals, and accelerometer readings, achieving up to 94.4% accuracy in two-stage classification and improving Cohen’s kappa values for sleep-stage differentiation [16,132,133,134]. CNNs effectively extract spatial patterns from raw sensor data, while RNNs, including Long Short-Term Memory (LSTM) and Gated Recurrent Unit (GRU) networks, capture temporal dependencies, enhancing sequential sleep-stage transitions [133]. Hybrid approaches combining CNNs for feature extraction and RNNs for temporal modeling have further improved accuracy while reducing misclassification errors caused by motion artifacts or sensor noise [132]. Additionally, AI-powered models can predict sleep efficiency, total sleep time, and disturbances, enabling personalized sleep improvement strategies [134]. Future work should refine these architectures by integrating attention mechanisms and transfer learning to enhance sleep-tracking reliability in consumer devices [18].

AI-driven sleep tracking raises significant ethical concerns, particularly regarding data privacy, informed consent, and potential medicalization of sleep [135,136]. The technology’s lack of transparency and potential biases pose challenges for user autonomy and fairness [135]. While wearable devices offer convenience, they often restrict users’ access to raw data, limiting understanding of analysis uncertainty and data reusability [137]. Ethical implications extend to vulnerable populations and third-party privacy [136]. Despite these concerns, AI in sleep medicine has the potential to transform patient care through improved screening, diagnosis, and treatment [138]. To address these challenges, the responsible development and implementation of sleep-tracking applications should focus on transparency, data protection, and user empowerment [135,136]. Balancing the benefits of AI-driven sleep tracking with ethical considerations is crucial for building trust in this emerging technology.

Beyond classification accuracy, AI is revolutionizing personalized sleep coaching. AI-powered applications now offer tailored recommendations on sleep hygiene, circadian rhythm optimization, and bedtime routines. Some systems also integrate with IoT-enabled smart home devices, adjusting lighting and temperature based on real-time sleep patterns. However, standardizing AI models across different devices and ensuring transparency in proprietary algorithms remain ongoing challenges.

Future work in addressing these challenges should focus on several key areas to enhance their integration into clinical practice. First, rigorous validation of AI and ML algorithms on diverse, multimodal clinical datasets is essential to ensure their reliability and accuracy in diagnosing and managing sleep disorders [139]. As AI continues to advance, it is crucial to develop standardized approaches for integrating AI with existing diagnostic tools, such as polysomnography (PSG), to improve clinical workflows and patient care [140]. Moreover, the application of Explainable AI (XAI) techniques will play a vital role in addressing the “black-box” problem by enhancing transparency and interpretability, ensuring that healthcare providers can trust and understand AI-driven recommendations [141,142]. Efforts should also focus on overcoming challenges related to data privacy, sensor accuracy, and the generalization of AI models across diverse populations [143]. Finally, collaboration between researchers, healthcare providers, and regulators is needed to create best practices and guidelines that ensure AI systems are validated, standardized, and ethically integrated into sleep medicine, ultimately facilitating more personalized, efficient, and accessible treatment options for sleep disorders [138,143].

### 7.5. Emerging Trends and Future Directions

The future of home sleep monitoring is shifting toward non-contact and more accurate solutions. Radar-based and radio-frequency (RF) sensors are emerging as alternatives to wearables and mattress-based trackers, offering passive and unobtrusive monitoring without requiring direct contact [62]. AI-driven sleep coaching applications are also evolving, integrating real-time adjustments based on individual sleep patterns and environmental factors.

Another key trend is the integration of sleep monitoring with smart home ecosystems. IoT-enabled devices allow sleep trackers to communicate with smart thermostats, lighting systems, and ambient noise controllers to optimize sleep conditions automatically. Additionally, cloud-based health platforms are facilitating remote sleep assessments and early interventions for sleep disorders. Addressing data security, interoperability, and regulatory compliance will be essential for widespread adoption.

### 7.6. Data Privacy and Security in Home Sleep Monitoring

Home-based sleep monitoring technologies offer significant benefits but raise critical privacy and security concerns. Implementing blockchain can enhance data security in remote health monitoring by ensuring decentralization, immutability, and transparency [144,145]. For in-home insomnia monitoring, efficient and secure transmission protocols are essential to protect sensitive health data [145]. The COVID-19 pandemic has accelerated the adoption of home monitoring technologies, underscoring the need for stringent safety, privacy, and regulatory measures, including Emergency Use Authorizations and compliance with privacy laws [135]. However, IoT-based sleep trackers remain vulnerable to security risks, such as third-party app exploitation, insecure communication channels, and cloud storage breaches. Strengthening security through regular software updates, user awareness programs, and social engineering prevention strategies can mitigate these risks [146]. Addressing these challenges is crucial for ensuring user trust, data protection, and regulatory compliance in home-based sleep monitoring systems.

### 7.7. Implications for Users and Researchers

For users, the increasing availability of home sleep monitoring technologies offers valuable insights into sleep health. However, it is essential to recognize their limitations. While these tools can track general sleep trends, they should not replace clinical evaluations for diagnosing conditions, like sleep apnea or REM behavior disorder. Future advancements in AI-driven sleep coaching could further enhance user experience by offering personalized interventions based on behavioral patterns.

For researchers, the challenge lies in scientific validation. Proprietary algorithms and a lack of standardization hinder independent verification. Open-source frameworks are needed to benchmark sleep-tracking accuracy and ensure clinical applicability. Future research should explore hybrid models that combine consumer convenience with clinical precision, ultimately improving the credibility of home-based sleep tracking.

## 8. Conclusions

The advancements in home sleep monitoring technologies have significantly improved accessibility and convenience for tracking sleep health. While smartphone apps, wearables, and smart mattresses offer valuable insights into sleep patterns, they still present challenges related to accuracy, clinical validation, AI transparency, and privacy. Although AI-enhanced consumer devices have made strides in sleep-stage classification, they remain less reliable than gold-standard PSG. The integration of multi-sensor fusion, AI-driven analytics, and IoT connectivity is paving the way for more refined and clinically relevant home sleep monitoring solutions.

The future of sleep technology lies in improving AI model transparency, standardizing validation metrics, enhancing sensor capabilities, and ensuring regulatory compliance for data privacy. Research efforts should focus on establishing benchmarks for AI-driven sleep staging accuracy, expanding clinical validation studies for wearables and smart mattresses, and enhancing integration with healthcare systems.

From the analyzed data, this review offers essential insights to help users and researchers select the most appropriate home sleep monitoring solutions to meet individual requirements. Based on our evaluation, wearables provide the best balance between accuracy, affordability, and usability, making them a suitable choice for general users and athletes. Smartphone apps are the most cost-effective but suffer from lower accuracy, making them more suitable for casual tracking rather than clinical use. Smart mattresses, while offering passive and comfortable monitoring, come with higher costs and limited clinical validation. Understanding these trade-offs allows users to make informed decisions when selecting a home sleep monitoring solution that aligns with their needs and expectations.

Despite these challenges, home sleep monitoring technology has the potential to bridge the gap between consumer health tracking and clinical diagnostics, making personalized sleep health insights more accessible to a broader population. As these technologies continue to evolve, they will play a crucial role in preventative healthcare, early sleep disorder detection, and optimizing overall well-being.

## Figures and Tables

**Table 1:** Evaluation criteria for sleep monitoring technologies.

Category	Evaluation Metric	Description
Device Classification	Wearables, smartphone apps, or smart mattresses	Categorized for comparison
Sleep Metrics	Sleep duration, efficiency, and disturbances	Accuracy in measuring key sleep parameters
Physiological Data	HRV, respiratory rate, and SpO_2_	Assessment of vital signs related to sleep health
Tracking Method	Actigraphy, ballistocardiography (BCG), sound analysis, and EEG-based sensors	The type of data collection mechanism used
Data Privacy and Security	Compliance with HIPAA, GDPR, and data encryption protocols	Evaluate how well the device protects user data
Cost and Affordability	Device purchase, subscription fees, and free	Financial accessibility of the technology

**Table 2:** Data visualization features by device type.

Device Type	Visualization Platform	Visualization Methods
Mobile Sleep Apps	Smartphone app	Interactive graphs, color-coded charts, trend analysis
Wearable Devices	Wearable screen, smartphone app	Bar/line graphs, color-coded stages, trend analysis
Smart Mattresses	Smartphone/tablet app	Segmented graphs, environmental data, trend tracking

**Table 3:** Overview of the comparison between PSG and actigraphy sleep monitoring.

Criteria	PSG (Polysomnography)	Actigraphy
Accuracy	High accuracy, gold standard for sleep disorder diagnosis.	Moderate accuracy, less precise in detecting sleep stages.
Data Collected	Multiple physiological signals (EEG, ECG, EMG, EOG, etc.).	Movement data (via accelerometer), often combined with sleep/wake status.
Sleep-Phase Detection	Precise detection of sleep stages (NREM, REM, etc.).	Limited to general sleep/wake detection, with basic phase detection.
Application	Diagnosing complex sleep disorders (e.g., sleep apnea, narcolepsy).	Long-term monitoring, behavioral sleep tracking, lifestyle intervention assessment.
Limitations	Requires a controlled environment (sleep lab), expensive, time-consuming.	Less accurate in diagnosing disorders, cannot detect complex physiological events.

**Table 4:** Overview of home-based sleep monitoring technologies.

Category	Technologies	Data Collected	Applications	Limitations
Smartphone Applications	Mobile apps	Movement, sound, heart rate (via sensor)	Basic sleep tracking, snoring, sleep quality	Limited precision, dependent on phone placement
Wearable Devices	Smartwatches, EEG headbands, chest straps	Movement, heart rate, EEG, SpO_2_	Sleep stages, heart rate, sleep apnea detection	Accuracy limitations, discomfort, restricted sleep-stage detection
Smart Mattresses	Smart mattresses, multi-sensor systems	Movement, respiration, heart rate, EEG	Comprehensive sleep data (stages, health, disturbances)	Complex integration, higher cost, potential privacy concerns

**Table 5:** Key features of smartphone apps for sleep monitoring.

Smartphone Apps	Key Features	Technology	Platform Rating and Number	AI Application	Device Integration
Sleep Cycle [34]	Sleep-stage tracking, smart alarm, snore detection, and movement.	Microphone, accelerometer	Android: 4.6 (206 K) iOS: 4.6 (2.6 K)	Uses patented AI	Yes
SleepScore [35]	Sleep tracking, sleep score, and sleep report.	Sonar technology	Android: 3.1 (902) iOS: 4.4 (7.3 K)	Uses patented AI	Yes
Sleep Monitor [36]	Track sleep talk, cycle, and analysis; smart alarm.	Not mentioned	Android: 4.6 (97 K) iOS: 4.5 (475)	No	No
Sleep as Android [37]	Monitor sleep cycle, sleep score, and sleep talk.	Accelerometer	Android: 4.5 (379 K)	No	Wearables, third-party apps
Snore Recorder [38]	Snore detection and sleep analysis.	Microphone	iOS: 4.5 (64)	No	Apple Health
ShutEye [39]	Sleep tracking, smart alarm, and snore detection.	Microphone	Android: 4.5 (86.9 K) iOS: 4.8 (316 K)	Yes	No
Pillow [40]	Sleep tracking, analysis, smart alarm, sleep aid, and insights.	Microphone, accelerometer	iOS: 4.4 (93.6 K)	No	Apple devices
SleepBot [41]	Sleep tracking, HR analysis, relaxing sound, sleep alerts, and timer.	Accelerometer	iOS: 4.6 (52)	Yes	iPhone
Sleep Tracker—Sleep Sounds [42]	Sleep tracking, insights, and goals. Sound therapy and smart alarm.	Accelerometer, gyroscope, microphone	Android: 4.6 (154 K) iOS: 4.8 (5.3 K)	Yes	Smart home devices, watches, voice assistance
SnoreLab [43]	Snore detection and sleep statistic.	Microphone	Android: 4.6 (47 K) iOS: 4.7 (53.8 K)	No	Apple Health
NapBot—Auto Sleep Tracker [44]	Sleep tracking, analysis, and history; HR monitor.	Accelerometer	iOS: 4.2 (6.2 K)	Yes	Apple Health
Sleep Details [45]	Sleep tracking, score, patterns, and insights.	Microphone, accelerometer	iOS: 4.4 (950)	Yes	Wearables, home devices, health apps, medical devices
iPhone Health [46]	Sleep, menstrual, activity, and medication tracking, mental health, Hearing test, Heart Health Monitoring, and ac.	Accelerometer, gyroscope	iOS: 3.0 (6.4 K)	No	Apple Watch, third-party devices
Sleepia [47]	Sleep analysis and programs, snore detection, environment sound analysis, and smart alarm.	Microphone	Android: iOS: 4.6 (119)	Yes	Apple Health
Mintal Tracker [48]	Sleep tracking, report, booster, aid, and talk recording; snore detection, alarm clock, and audio tracks.	Microphone	Android: 3.5 (609) iOS: 4.8 (38 K)	Yes	Apple Health
Alarmy [49]	Sleep analysis and sound; snore detection.	Microphone, accelerometer, camera	Android: 4.5 (1.78 M) iOS: 4.8 (199.2 K)	No	No
Sleep time [50]	Sleep analysis, history, smart alarm, and soundscapes.	Accelerometer	iOS: 4.7 (5.3 K)	No	Apple Health
Sleepace [51]	Sleep monitoring, aid, reports, and tips; smart alarm.	Microphone, motion sensors	iOS: 1.9 (27)	Yes	Apple Health
Rise [52]	Sleep tracking: duration, quality, stage, and deficit.	Accelerometer, microphone	Android: 4.2 (5.8 K) iOS: 4.6 (25.7 K)	AI and ML	Wearables, home devices, health apps
Sleepzy [53]	Sleep tracking: pattern, quality, and debt analysis; smart alarm.	Microphone	iOS: 4.3 (22.4 K)	AI to generate music	Apple Health and watch
SlumberCycle [54]	Sleep tracking, recording, and aid; smart alarm.	Smartphone’s built-in sensors	Android: 3.9 (6.7 K)	No	No

**Table 6:** Key features of wearable sleep-tracking devices.

Smartwatch	Sleep Features Monitored	Technology (Sensors)	AI Usage	Visualization Platform	Other Features
Apple Watch Ultra 2 [65]	Track sleep stages, breathing disturbances, sleep duration	Accelerometer, HR sensor, SpO_2_, Skin temp, Mic	Machine learning for sleep-stage detection	Apple Health, Sleep app	36 h battery, ECG, dual-frequency GPS
Apple Watch Series 9 [66]	Sleep stages, HR, SpO_2_, Resp. rate	Accelerometer, HR sensor, SpO_2_, Mic	Machine learning for sleep tracking	Apple Health, Sleep app	18 h battery, ECG, fast charging
Samsung Galaxy Watch 6 [67]	Sleep stages, HR, SpO_2_, snoring detection, HRV	Accelerometer, BioActive sensor (HR, SpO_2_), Mic	AI-driven sleep coaching, snore pattern analysis	Samsung Health	3-day battery, body composition tracking
Fitbit Sense 2 [68]	Sleep score, stages, HR, SpO_2_, Skin temp, HRV	Accelerometer, HR sensor, SpO_2_, Skin temp	AI-driven stress and sleep insights	Fitbit app (premium for full insights)	6-day battery, EDA stress tracking
Garmin Venu 3 [69]	Sleep score, stages, HR, SpO_2_, Resp. rate, HRV	Accelerometer, HR sensor, SpO_2_, Barometer	AI-based Body Battery for recovery analysis	Garmin Connect	14-day battery, advanced fitness tracking
Garmin Fenix 7 Pro [70]	Sleep score, HRV, stages, SpO_2_, Resp. rate	HR sensor, SpO_2_, Barometer, Altimeter	AI-based Body Battery and recovery tracking	Garmin Connect	Rugged design, solar charging option
Withings ScanWatch 2 [71]	Sleep stages, HR, SpO_2_, sleep apnea detection	ECG, HR sensor, SpO_2_, motion sensor	AI-based apnea detection, deep sleep analysis	Withings Health Mate	30-day battery, hybrid smartwatch
Google Pixel Watch 2 [72]	Sleep stages, HR, SpO_2_, Resp. rate	Fitbit sensors: HR, SpO_2_, accelerometer	Fitbit AI for sleep trends and wellness coaching	Fitbit app, Google Health	24-h battery, Wear OS 4
Huawei Watch GT 3 [73]	Sleep stages, HR, SpO_2_, Resp. rate	TruSleep 3.0, HR sensor, SpO_2_, accelerometer	AI sleep coaching, personalized insights	Huawei Health	14-day battery, built-in GPS
Polar Ignite 3 [74]	Sleep score, sleep stages, Nightly Recharge	Accelerometer, HR sensor, SpO_2_	AI Nightly Recharge analysis	Polar Flow	5-day battery, advanced training metrics
Amazfit Bip 5 Unity [75]	Sleep stages, HR, SpO_2_	BioTracker PPG 4.0 sensor, accelerometer	AI-based sleep trends analysis	Zepp app	10-day battery, lightweight design
Suunto 9 Peak Pro [76]	Sleep stages, HR, SpO_2_	HR sensor, SpO_2_, Altimeter, Barometer	AI-based sleep and recovery tracking	Suunto App	21-day battery, ultra-durable design
ASUS VivoWatch 5 plus [77]	Sleep stages, HR, SpO_2_	ECG, HR sensor, SpO_2_, accelerometer	AI-based algorithm maximizes battery life	HealthConnect App	14-day battery, built-in GPS, water resistant
Oppo Watch [78]	Sleep score, sleep stages, HR, SpO_2_	PPG HR sensor, ECG, accelerometer, gyroscope, Barometer	AI imaging algorithm, AI-based sleep	Oppo Health App	21-day battery, dual-chip endurance system
Mi Watch [79]	Sleep score, sleep stages, HR, SpO_2_	PPG HR sensor, ECG, accelerometer, gyroscope, Barometer	AI-based algorithm battery life extension	Mi Fitness App	16-day battery, built-in GPS
Maimo Watch [80]	Sleep score, sleep stages, HR, SpO_2_	PPG HR sensor, ECG, accelerometer, gyroscope, Barometer	AI-based sleep, Running Competitor	Maimo fit App	10-day battery, built-in Alexa, water resistant

**Table 7:** Smart mattresses with sleep monitoring features.

Smart Mattresses	Key Sleep Monitoring Features	Other Features	Technology (Sensors)	AI Application	Sleep Visualization Method
Eight Sleep Pod Pro Cover [85]	Sleep tracking and temperature control.	Temperature Control, wake-up technology	Embedded sensors, active grid layer	Yes	Dedicated app
Sleep Number 360 [86]	Monitors sleep quality, HR, breathing, movement, and pressure.	Pressure Adjustments, Automatic Positioning, Temperature Control	Embedded sensors	Yes	SleepIQ app
ReST Bed [87]	Tracks sleep position, movement, and pressure.	Customizable firmness for 5 body zones	Smart fabric sensors	Yes	ReST Bed™ app
Withings Sleep Analyzer [88]	Sleep apnea detection, snore detection, sleep analysis, and heart rate monitoring.	Automatic Wi-Fi Sync	Pneumatic sensor, ballistocardiography	Yes	Withings Health Mate app
Tempur-Pedic LuxeBreeze with Ergo^®^ Smart Base [89]	Tracks various sleep metrics: efficiency, duration, stages, etc.	Adjustable base, Pressure Relief, cooling tech	Sensors embedded in the Ergo^®^ Smart Base	Yes	Tempur-Ergo Smart Base app
Bryte Balance Smart Bed [90]	Tracks sleep patterns, stages, duration, and has Sleep Concierge.	AI adjusts firmness, Zero Gravity Position	Embedded sensors	Yes	Bryte app
Whizpad Mattress [91]	Tracks movement and sleep activity.	Pressure Redistribution, Early Leave-Bed Alerts	Pressure sensors	Yes	Whizpad app
NordicTrack Sleep Mattress [92]	Tracks HR, respiration, awakenings, bed exits, and sleep metrics.	Sleep coaching, smart alarm	iFit Sleep HR sensor	No	iFit Sleep app
ERA Smart Layer [93]	Sleep pattern and heat rate and breathing analysis.	Active Spinal Alignment, Multizone Relaxation, Thermal Regulation	BCG sensors	Yes	ERA App

**Table 8:** Sleep monitoring technologies for different user scenarios.

User Scenario	Recommended Technology	Key Features	Limitations
General Users	Smartphone apps, wearables	Cost-effective, widely available	Lower accuracy in sleep staging
Sleep Disorders	FDA-approved wearables, smart mattresses	Sleep apnea detection, long-term tracking	Less accurate than PSG
Athletes and Recovery	Smartwatches, smart mattresses	HRV analysis, deep sleep tracking, recovery monitoring	Smart mattresses are expensive, AI-based insights need validation
Elderly	Smart mattresses, non-contact sensors	Passive monitoring, caregiver alerts	High cost, limited health integration
Mental Health Applications	Wearables, smartphone apps	HRV-based stress analysis, AI sleep coaching	AI interpretation of HRV–sleep relationship still evolving
Children’s Sleep Monitoring	Smart baby monitors, wearables	Safety alerts, sleep duration tracking	Privacy concerns, limited sleep-stage accuracy

**Table 9:** Cost–benefit comparison of home-based sleep monitoring technologies.

Device Type	Cost (USD)	Accuracy	Health Benefits	Best For
Smartphone Apps	Free–USD50	Low	Basic sleep tracking, lack physiological data	Budget-conscious users
Wearables	USD100–USD500 + subscription	Medium–High	Track HRV, SpO_2_, sleep stages, AI coaching	Athletes, sleep-conscious individuals
Smart Mattresses	USD500–USD3000 + subscription	Medium	Passive tracking, sleep position adjustment	Long-term users, couples, hands-free use

**Table 10:** Device data collection across categories.

Device Category	Device	Data Category			
		Sleep Metrics	Physiological Data	Movement Data	Environmental Data
Smartphone apps	Sleep Cycle [34]	✔		✔	✔
	SleepScore [35]	✔	✔	✔	
	Sleep Monitor [36]	✔	✔	✔	
	Sleep as Android [37]	✔		✔	✔
	Snore Recorder [38]	✔			✔
	ShutEye [39]	✔	✔	✔	✔
	Pillow [40]	✔	✔	✔	
	SleepBot [41]	✔		✔	✔
	Sleep Tracker—Sleep Sounds [42]	✔		✔	✔
	SnoreLab [43]	✔			✔
	NapBot—Auto Sleep Tracker [44]	✔	✔	✔	
	Sleep Details [45]	✔	✔	✔	
	iPhone Health [46]	✔	✔	✔	
	Sleepia [47]	✔	✔	✔	✔
	Mintal Tracker [48]	✔		✔	✔
	Alarmy [49]	✔		✔	
	Sleep time [50]	✔		✔	✔
	Sleepace [51]	✔	✔	✔	
	Rise [52]	✔	✔	✔	
	Sleepzy [53]	✔		✔	✔
	SlumberCycle [54]	✔		✔	✔
Smartwatches	Apple Watch Ultra 2 [65]	✔	✔	✔	✔
	Apple Watch Series 9 [66]	✔	✔	✔	✔
	Samsung Galaxy Watch 6 [67]	✔	✔	✔	✔
	Fitbit Sense 2 [68]	✔	✔	✔	✔
	Garmin Venu 3 [69]	✔	✔	✔	
	Garmin Fenix 7 Pro [70]	✔	✔	✔	
	Withings ScanWatch 2 [71]	✔	✔	✔	
	Google Pixel Watch 2 [72]	✔	✔	✔	✔
	Huawei Watch GT 3 [73]	✔	✔	✔	
	Polar Ignite 3 [74]	✔	✔	✔	
	Amazfit Bip 5 Unity [75]	✔	✔	✔	
	Suunto 9 Peak Pro [76]	✔	✔	✔	
	ASUS VivoWatch 5 plus [77]	✔	✔	✔	
	Oppo Watch [78]	✔	✔	✔	
	Mi Watch [79]	✔	✔	✔	
	Maimo Watch [80]	✔	✔	✔	
Smart mattresses	Eight Sleep Pod Pro Cover [85]	✔	✔	✔	✔
	Sleep Number 360 [86]	✔	✔	✔	✔
	ReST Bed [87]	✔	✔	✔	✔
	Withings Sleep Analyzer [88]	✔	✔	✔	✔
	Tempur-Pedic LuxeBreeze with Ergo^®^ Smart Base [89]	✔	✔	✔	✔
	Bryte Balance Smart Bed [90]	✔	✔	✔	✔
	Whizpad Mattress [91]	✔	✔	✔	
	NordicTrack Sleep Mattress [92]	✔	✔	✔	✔
	ERA Smart Layer [93]	✔	✔	✔	✔
